# Bortezomib prevents cytarabine resistance in MCL, which is characterized by down-regulation of dCK and up-regulation of SPIB resulting in high NF-κB activity

**DOI:** 10.1186/s12885-018-4346-1

**Published:** 2018-04-25

**Authors:** Catja Freiburghaus, Venera Kuci Emruli, Angelica Johansson, Christian Winther Eskelund, Kirsten Grønbæk, Roger Olsson, Fredrik Ek, Mats Jerkeman, Sara Ek

**Affiliations:** 1 Department of Immunotechnology, Lund University, Lund, Sweden; 2 Department of Haematology, Rigshospitalet, Copenhagen, Denmark; 3 Department of Experimental Medical Science, Chemical Biology & Therapeutics, Lund University, Lund, Sweden; 4 Department of Oncology, Lund University, Lund, Sweden

**Keywords:** Cytarabine, DCK, MCL, NF-κB, SPIB

## Abstract

**Background:**

The addition of high-dose cytarabine to the treatment of mantle cell lymphoma (MCL) has significantly prolonged survival of patients, but relapses are common and are normally associated with increased resistance. To elucidate the mechanisms responsible for cytarabine resistance, and to create a tool for drug discovery investigations, we established a unique and molecularly reproducible cytarabine resistant model from the Z138 MCL cell line.

**Methods:**

Effects of different substances on cytarabine-sensitive and resistant cells were evaluated by assessment of cell proliferation using [methyl-14C]-thymidine incorporation and molecular changes were investigated by protein and gene expression analyses.

**Results:**

Gene expression profiling revealed that major transcriptional changes occur during the initial phase of adaptation to cellular growth in cytarabine containing media, and only few key genes, including SPIB, are deregulated upon the later development of resistance. Resistance was shown to be mediated by down-regulation of the deoxycytidine kinase (dCK) protein, responsible for activation of nucleoside analogue prodrugs. This key event, emphasized by cross-resistance to other nucleoside analogues, did not only effect resistance but also levels of SPIB and NF-κB, as assessed through forced overexpression in resistant cells. Thus, for the first time we show that regulation of drug resistance through prevention of conversion of pro-drug into active drug are closely linked to increased proliferation and resistance to apoptosis in MCL. Using drug libraries, we identify several substances with growth reducing effect on cytarabine resistant cells. We further hypothesized that co-treatment with bortezomib could prevent resistance development. This was confirmed and show that the dCK levels are retained upon co-treatment, indicating a clinical use for bortezomib treatment in combination with cytarabine to avoid development of resistance. The possibility to predict cytarabine resistance in diagnostic samples was assessed, but analysis show that a majority of patients have moderate to high expression of dCK at diagnosis, corresponding well to the initial clinical response to cytarabine treatment.

**Conclusion:**

We show that cytarabine resistance potentially can be avoided or at least delayed through co-treatment with bortezomib, and that down-regulation of dCK and up-regulation of SPIB and NF-κB are the main molecular events driving cytarabine resistance development.

**Electronic supplementary material:**

The online version of this article (10.1186/s12885-018-4346-1) contains supplementary material, which is available to authorized users.

## Background

Mantle cell lymphoma (MCL) is an aggressive B-cell lymphoma, molecularly defined by the translocation of *CCND1* [1]. The malignant cells harbor a number of molecular abbreviations such as overexpression of SOX11 [2] and constitutive activation of the nuclear factor-κB (NF-ĸB) pathway [3]. The NF-ĸB pathway regulates a number of genes involved in apoptosis, cell adhesion, proliferation and tissue remodeling. Especially, relapsed MCL has increased activity of the pathway which most likely has a key role in maintaining tumour cell viability and drug resistance, through overexpression of several anti-apoptotic proteins [4, 5].

Traditionally, MCL was characterized by initial sensitivity to standard chemotherapy followed by relapse, and unfavorable outcome [6, 7]. However, addition of high-dose cytarabine treatment as part of the induction therapy has resulted in great improvement in survival in subgroups of MCL patients [8]. Cytarabine (ara-C, cytosine arabinoside) is a deoxycytidine nucleoside analogue, an S-phase specific anti-metabolite, which is used in modern MCL combinatorial treatment protocols [9]. High-dose cytarabine is effective due to the improved retention of ara- CTP by target cells [10], but likewise toxic, causing mainly hematological side effects. Thus, understanding the molecular mechanism(s) responsible for resistance, identifying predictive markers for resistance and/or sensitizing agents, would be of great clinical value.

Cytarabine is a prodrug, which first needs to be transported across the plasma membrane, and secondly become activated through phosphorylation. Transportation of nucleosides and nucleoside analogues across the plasma membrane is mediated by transporter proteins belonging to the solute carrier families 28 and 29 (*SLC28* and *SLC29*). *SLC28* genes encode the three members of the concentrative nucleoside transporter (CNT) family, while the four members of equilibrative nucleoside transporter (ENT) proteins are encoded by *SLC29* genes [11]. Both ENT and CNT recognise most of the nucleoside analogues used for cancer therapy and as such they are interesting targets for further studies. For most of the nucleoside analogues commonly used for anti-cancer therapy, the first phosphorylation step is catalysed by deoxycytidine kinase (dCK). Both de novo resistance and acquired resistance to cytarabine, including cross-resistance to other nucleoside analogues, have been linked to down-regulation of dCK on gene and protein level [12–14].

Today, there are many treatment alternatives available for relapsed or recurrent MCL patients but only little information available on which patients that would benefit from each alternative. Thus, the aims of the present study were to (i) characterize the mechanisms of cytarabine resistance in MCL, (ii) identify drugs suitable for treatment of relapsed/recurrent MCL patients treated with Ara-C and (iii) to suggest preventive measurements based on in vitro-model data.

To do so we have established a unique MCL resistant model in which cytarabine resistance repetitively and molecularly reproducibly can be induced in a highly controlled manner. Using molecular profiling, we show that down-regulation of the dCK protein is key to development of resistance. The cellular model, representing three stages of resistance development (naïve sensitive, exposed sensitive and resistant) was further characterized using gene expression analysis and functional analysis. Key gene changes, including upregulation of the transcription factor *SPIB* was identified. We further show that similar to relapsed/recurrent MCLs, the resistant cells are not only defined by the lack of dCK and increased SPIB, but also high levels of NF-κB.

Functional screens using (i) chemotherapeutics or (ii) epigenetic regulators were used to identify drugs with potential cross-resistance and/or sensitivity, and to select individual epigenetic candidate drugs for sensitisation of cytarabine-resistant cells. Co-treatment with bortezomib and cytarabine prevented resistant development, but could not overcome resistance once dCK was abolished.

The importance of dCK for response to therapy was confirmed by the analysis of primary MCLs where 97% of the patients have high or intermediate dCK at diagnosis.

## Methods

### Cell culture

The MCL cell line Z138 was purchased from ATCC (Manassas, VA, USA). Both untreated and resistant cell lines were cultured in R10 medium (RPMI 1640 (HyClone Laboratories, Logan, UT, USA) supplemented with 10% fetal bovine serum (Invitrogen, Carlsbad, CA, USA) and 1% 2 mM L-glutamine (Invitrogen)).

### Reagents

Cytarabine (147–94-4, Pfizer, New York, NY, USA) was aliquoted and stored at 4 °C with bulk concentrations of 411 mM. SCREEN-WELL® Epigenetics library (BML-2836, Enzo LifeSciences Inc., Farmingdale, NY, USA) and Chemotherapeutic Agent Library (L1500, Selleck, Munich, Germany) were stored at − 80 °C until use. Substances used for proliferation studies including bortezomib (2204S, Cell Signaling Technologies), lenalidomide (PCID-216326 Santa Cruz Biotechnology, USA), apicidin (A8851, Sigma Aldrich), belinostat (PXD101), M-344 (M5820, Sigma Aldrich), oxamflatin (O3139, Sigma Aldrich, St. Louis, MO, USA), scriptaid (S7817, Sigma Aldrich), trichostatin A (T1952, Sigma Aldrich) and vorinostat (SAHA MK0683, Selleck) were dissolved in DMSO (Sigma Aldrich), aliquoted and stored at − 80 °C until use.

### Establishment of resistant sub-clones

The first resistant sub-clone defined as Z138 Cytarabine Resistant (Z138-CytR) was established by continuous exposure of wild type Z138 Cytarabine Naïve Sensitive cells (Z138-CytNS) to increasing concentrations (0.005–0.3 μM) of cytarabine. Using this model, we could identify the approximate time to resistance development, and utilize this information for developing a novel time-controlled cytarabine resistant model, described below.

Z138-CytNS, with viability above 85%, were exposed to 0.005 μM cytarabine and kept at log phase (1–2 × 10^6^ cells/ml). Concentrations were increased two- or ten-fold, and samples for immunoblotting were taken when viability reached above 85%. When reaching a concentration of 0.2 μM cytarabine, cells were expanded and frozen as a cell biobank, a sub-clone called Z138 Cytarabine Exposed Sensitive (Z138-CytES). This cell biobank could then be used for further cytarabine exposure experiments and the establishment of a Cytarabine Resistant 21 days (Z138-CytR21) sub-clone.

### Effect of cytarabine on sensitive and resistant cell lines

Cells were seeded in a 48 well plate and incubated with 0, 0.5, 5 and 50 μM of cytarabine at 37 °C (5% CO_2_) for 24–96 h. Duplicates from each concentration were counted in an automatic cell counter (Countess™, Invitrogen) at each time point, and trypan blue exclusion method was used to monitor viability.

### Assessment of cell proliferation by [methyl-^14^C]-thymidine incorporation

Cells were seeded in a Cytostar-T 96 well plate (Perkin Elmer, Waltham, MA, USA) and cultured for up to 72 h in presence of 0.5 μCi/ml [methyl-^14^C]-thymidine (PerkinElmer). Cell proliferation was measured at indicated time-points using a Wallac 1450 MicroBeta liquid scintillation counter (Perkin Elmer). Prior to all measurements, cells were centrifuged to allow contact to the scintillation liquid.

### Re-introduction of dCK into dCK negative resistant cells

To assess the importance of dCK in relation to resistance, the protein was transiently re-introduced into resistant cells. The Amaxa protocol (Amaxa Biosystems Cologne, Germany) for nucleofection of suspension cell lines was followed, using program CM-138 and Cell Line Nucleofector Solution SF (Amaxa Biosystems). For the re-introduction experiments, 2.5 × 10^6^ cells were mixed with 2 μg of OmicsLink™Expression Clone for dCK (EX-C0081-M46, vector information can be found in Additional file 1) in each reaction and a GFP vector was used as a positive control (both from GeneCopoeia, Germantown, MD, USA).

### Gene expression analysis

Triplicate cultures of cells were harvested at different time-points and lysed in TRIzol (Invitrogen). Preparation of tRNA was performed as previously described [15]. Gene expression was assessed using Affymetrix Human Transcriptome Array 2.0 (HTA 2.0; Affymetrix Inc., Santa Clara, CA, USA), and acquired data was pre- processed at the SCIBLU Genomics Centre (Lund University, Sweden) involving quality control and normalization, using the Expression Console software (Affymetrix Inc). Normalized and log^2^ transformed data was imported into Qlucore Omics Explorer 3.0 (Qlucore AB, Lund, Sweden) for statistical analysis. For confirmation of mRNA expression in different samples, TaqMan probe-based RT-PCR was performed, using TaqMan® Fast Universal PCR Master Mix (Applied Biosystem, Waltham, MA, USA) and the TaqMan assay Hs01040726_m1 (dCK, Applied Biosystem) and Hs00162150_m1 (SPIB, Applied Biosystem). 18S (Hs99999901_s1, Applied Biosystem) was used as reference gene. All data were analyzed using the 7500 software v2.0.5 (Applied Biosystem). Functional annotation of individual genes was obtained from NCBI/Gene (http://www.ncbi.nlm.nih.gov/gene), GeneCards (http://www.genecards.org/) or UniProt (http://www.uniprot.org/).

### Library preparation, hybridization capture and MPS sequencing

DNA from Z138-CytNS, Z138-CytES and Z138-CytR cells were purified using RNeasy Plus Mini Kit (Qiagen, Hilden, Germany) and thereafter quantified using the Qubit system (Life Technologies, Carlsbad, CA, USA). Two μg of DNA were fragmented using the Covaris S2 Ultrasonicator (Covaris, Woburn, MA, USA) and DNA fragments from 64 target genes, including TP53 were captured using SureselectXT Custom 3–5.9 Mb library kit (Agilent Technology, Santa Clara, CA, USA). Before capture, eight samples were pooled, and the molarity of the pooled library was determined based on and DNA fragment size distribution measured on a Bioanalyzer (Agilent) and concentration measured by Qubit. Sequencing was performed on the Illumina HiSeq 2500 (Illumina, San Diego, CA, USA) with 2 × 101 bp paired end reads.

### Analysis of sequencing data

Picard Extract IlluminaBarcodes and IlluminaBasecallsToSam (https://broadinstitute.github.io/picard/) was used for format conversion and demultiplexing of raw Illumina sequencing data and sequence reads were aligned to the human reference genome hs37d5ss (1000 genome with decoy sequences) using Novoalign (http://www.novocraft.com). Picard MarkDuplicates were used to identify and exclude PCR duplicates in subsequent analyses and quality scores were recalibrated and indels realigned using the Genome Analysis Tool Kit (GATK) [16]. GATK UnifiedGenotyper with a call confidence cutoff of 10 were used to identify genetic variants and genotypes. Variants were annotated for their effect on protein coding transcripts using snpEff and Annovar using RefSeq reference transcripts. Bases of coding exons and 20 bp of adjacent introns were covered by at least 30 reads and variants affecting coding exons and 20 bp of adjacent introns were evaluated for pathogenicity.

### Silencing of SPIB in the resistant cell line

Cell Line Nucleofector Solution SF was used with program CM-138 following the Amaxa protocol for suspension cell lines (Amaxa Biosystems). Cells were mixed with 1000 nM of siRNA (Ambion, Austin, TX, USA) or a scrambled sequence. GFP-producing plasmid was used as control for the transfections (Amaxa Biosystems).

### Effect of bortezomib on resistance development

Based on the set up presented above, Z138-CytES cells were co-treated with 0.3 μM of cytarabine and 0.001 or 0.01 μM of bortezomib during the 21 days expected for resistance to develop. A positive control with only 0.3 μM of cytarabine was grown in parallel (as visualized in Fig. 10c). Z138-CytNS cells and Z138-CytR cells were subjected to the same treatment during the same period. Lysates for immunoblotting were sampled continuously during the treatment period and after completed treatment. After 21 days of treatment, proliferation in cytarabine containing medium was assessed by [methyl-^14^C]-thymidine incorporation as previously described.

### Immunoblotting

Cells were harvested for western blot and lysed on ice with lysis buffer (1% NP40 (Sigma Aldrich) in PBS supplemented with 1× complete protease inhibitor (Roche Applied Sciences, Indianapolis, IN, USA)) for 30 min followed by centrifugation at 4 °C, 1300 rpm for additional 30 min. Supernatants consisting of protein lysates were collected and protein concentrations were measured using a bicinchoninic acid kit (Sigma Aldrich). For western blot, 25 μg protein was loaded on a Bis-Tris gel (Life Technologies, Carlsbad, USA). Following electrophoresis using an XCell Surelock Mini- Cell system (Life Technologies), the proteins were immediately blotted onto a PVDF membrane using program P3 on the iBlot Dry Blotting System (Life Technologies). The membranes were then blocked for 60 min with 5% milk in PBS, before incubation with primary antibodies targeting dCK (TA502698, OriGene Technologies, Rockville, MD, USA), ENT1 (ab 11,337–1-AP, Proteintech, Chicago, IL, USA), SPIB (Cell Signaling), NF- κB (D14E12, Cell Signaling), IκBα (44D4, Cell Signaling) and/or GAPDH (G8795, Sigma Aldrich). Horseradish peroxidase-conjugated rabbit anti-mouse immunoglobulin (P0260, Dako, Glostrup, Denmark) and swine anti-rabbit immunoglobulin (P0217, Dako) were used as secondary antibodies. Protein levels were visualized in a ChemiDoc™ MP Imaging System (Bio-Rad Laboratories, Hercules, CA, USA) with SuperSignal West Femto Maximum Sensitivity Substrate (ThermoFisher Scientific). Quantification of the results was performed using the Image Lab software (Version 5.2.1, Bio-Rad Laboratories).

### Screening of compound libraries and validation of selected drugs

Z138-CytNS or Z138-CytR cells were seeded in a Cytostar-T 96 well plate as previously described, and treated with different concentrations of chemotherapeutic and epigenetic drugs. Non-treated cells re-suspended in R10 medium were considered as R10 controls, and DMSO (0.01%) treated cells as vehicle controls. Proliferation was measured 0, 24 and 48 h after addition of chemotherapeutic and/or epigenetic drugs. To evaluate the additive effect of epigenetic drugs to cytarabine, 50 μM cytarabine was added 6 h after pre-incubation of cells with epigenetic compounds.

### Patients, cohorts and treatment protocols

Materials from patients included in the Nordic Lymphoma Group MCL2 and MCL3 trials at hospitals in Sweden, Finland, Norway and Denmark, were selected for TMA construction as previously described [17]. The treatment protocols for MCL2 and MCL3 both include high-dose cytarabine, rituximab and ASCT as previously described [9].

### Immunohistochemistry staining and digital scoring

Immunohistochemistry was performed as previously described [2]. The sections were stained for dCK (TA502698; OriGene Technologies) and visually analysed using a Nikon ECLIPSE 80i microscope (Nikon Instruments Inc., Melville, NY, USA) at a magnification of 20× (Plan Flour 20× DIC M/N2, Nikon) with a numeric aperture of 0.5. Images were captured using a Nikon DS-U2/L2 USB (Nikon) camera, and NIS Elements BR 3.10 (Nikon) as acquisition software. For digital scoring, dCK stained slides were scanned at an absolute magnification of 20× (resolution of 0.493 μm per pixel) and digitally scored using HALO™ (Indica Labs, Corrales, NM, USA). Positive areas (tumour) and negative areas (stroma) were separated and quantified based on a pattern recognition algorithm in the HALO platform. Image analysis based on RGB (red, green, blue) spectra was used to detect all cells by counterstaining with hematoxylin (blue). All analysis settings including thresholds set for weak, intermediate and strong nuclei staining were maintained throughout the whole study (Additional file 2: Table S1).

## Results

### Establishment of reproducible cytarabine resistant sub-clones from the MCL cell line Z138

**Fig. 1:**
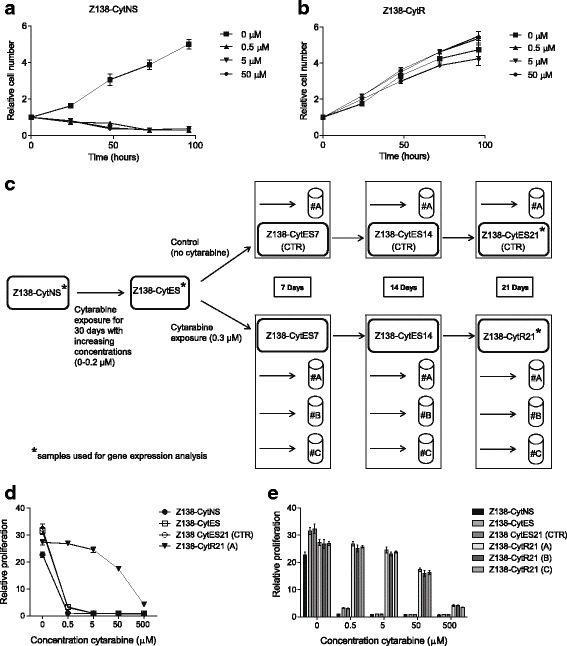
Functional evaluation of established Z138 sub-clones. Growth of (**a**) wild type, cytarabine naive sensitive (Z138-CytNS) cells and (**b**) an in-house developed cytarabine resistant (Z138-CytR) clone in presence of different concentrations of cytarabine, was assessed by trypan blue exclusion method at indicated time-points. Each data point represents a mean value of duplicates, and error bars show SEM. The data were normalized to the 0-h time-point. **c** Schematic overview of the established Z138 sub-clones. Z138-CytNS cells exposed to increasing concentrations (0–0.2 μM) of cytarabine for 30 days were expanded and frozen as a cell biobank named cytarabine exposed sensitive (Z138-CytES). The frozen cell biobank was thawed and used for establishment of a cytarabine resistant (Z138-CytR21) sub-clone, by exposing cells to 0.3 μM cytarabine for a period of 21 days. Biological replicates indicated as #A-C were included. In parallel, Z138-CytES cells cultured in absence of cytarabine for indicated days were used as controls (CTR). * marks the samples used for gene expression analysis (presented in Fig. 3a). **d** Assessment of cell proliferation by incorporation of [methyl-14C]-thymidine indicates that Z138-CytR21 cells remain unaffected until a concentration of 50 μM cytarabine is reached, as also illustrated by (**e**) biological replicates. Each data point represents a mean value of triplicates, and error bars show SEM. The data, representing the 48-h time-point, are normalized to the time-point 0 h

The Z138 cell line is originally derived from a MCL patient with blastoid transformation, and cells carry the 11;14 translocation and overexpress cyclin D1 [18]. The untreated Z138 cell line, hereafter referred to as Z138-CytNS (Z138 Cytarabine Naïve Sensitive) is highly sensitive toward cytarabine and does not survive concentrations at, or above 0.5 μM (Fig. 1a).

Z138-CytNS cells were exposed to increasing concentrations (0.005–0.3 μM) of cytarabine to investigate and define the number of days required to induce resistance upon continuous exposure. After approximately 60 days, a highly cytarabine resistant sub-clone, Z138-CytR, was established growing at an unaffected rate in up to 50 μM cytarabine (Fig. 1b).

With this information at hand, new cell cultures were initiated with the aim to establish a reproducible model where resistance could be induced in a time-controlled manner from a permanent source of viable frozen pre-exposed cells. Thus, Z138-CytNS cells were exposed to increasing concentrations of cytarabine and when reaching high viability (> 85%) at 0.2 μM, cells were expanded, without addition of cytarabine, and frozen to establish a large and renewable pool of cells with defined time to resistance development. From this pool of frozen cells (referred to as Z138-CytES), later experiments showed that complete resistance (defined as unaffected proliferation at the 50 μM cytarabine concentration) reproducibly can be achieved in ~ 21 days upon exposure to 0.3 μM cytarabine. To be able to investigate the difference in molecular response to cytarabine exposure and the molecular signature related to resistance development, control cells were cultivated in parallel without cytarabine during the last 21 days. A schematic representation of the experimental set-up and designation of samples at the different time-points of cytarabine exposure is shown in Fig. 1c. Comparison of cytarabine resistance in control (Z138-CytES21) and long-term cytarabine exposed cells (Z138-CytR21) showed that although control cells can be kept viable in 0.2 μM cytarabine (data not shown), they do not proliferate (Fig. 1d). Further comparison of replicates (*n* = 3) of cytarabine exposed cells and control cells at different time-points show a reproducible resistance of cells up to 50 μM, but loss of proliferation at 500 μM cytarabine (Fig. 1e).

As p53 status is a prognostic factor for patients treated with cytarabine-containing regimens [19], the TP53 status was investigated in both untreated and cytarabine-treated cells. All samples were shown to contain only TP53 WT alleles (data not shown). Proliferation was also investigated and shown to be similar in all three stages of resistance development (Additional file 2: Figure S1).

### Decreased expression of dCK and ENT during adaptation and development of resistance

**Fig. 2:**
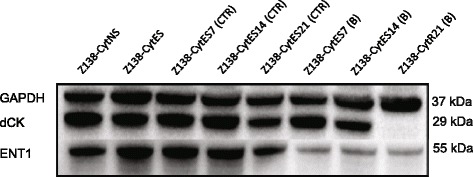
Assessment of dCK and ENT1 protein expression. Representative western blot analysis of indicated proteins in Z138 sub-clones. CytNS: cytarabine naive sensitive, CytES: cytarabine exposed sensitive, CytR21: cytarabine resistant, CTR: control, B: replicate B. GAPDH was used as loading control

It is previously known that cytarabine is dependent on the transport protein ENT1 to cross the cell membrane [20], and the dCK enzyme for phosphorylation into the active ara-CTP [21]. To evaluate the hypothesis that one of these important components is involved in the resistance development in MCL, we investigated the expression of the two proteins at various time-points of cytarabine exposure. Results show that ENT1 levels start to drop after approximately 7 days of exposure to 0.3 μM cytarabine, Z138-CytES7 (Fig. 2), but no further decrease is seen as the resistance fully develop (Z138-CytES14 compared to Z138-CytR21). Analysis of dCK show that only minor down-regulation is seen during the continuous exposure to cytarabine, but an abrupt abolishment of dCK co-occurs with resistance development (Z138-CytES14 compared to Z138-CytR21, Fig. 2). This is consistent in all three parallel experiments (Additional file 2: Figure S2).

**Fig. 3:**
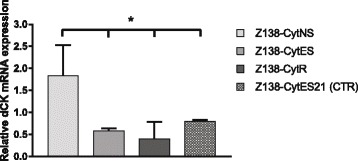
Verification of dCK mRNA expression in gene microarray samples. The relative mRNA expression of dCK was assessed in the different Z138 subclones using TaqMan probe based RT-PCR and revealed down-regulation in both Z138-CytES and Z138-CytR cells compared to Z138-CytNS cells. The data are normalised to the S18 reference gene, and the reference sample, replicate A of the Z138-CytNS for each run. Bars represent relative quantity ± SEM of three technical replicates. * = *p* < 0.05 with statistical significance determined using the Holm-Sidak method, with alpha = 0.05. All significance is compared with Z138-CytNS. Each row was analyzed individually, without assuming a consistent SD

Recovery studies, where Z138-CytR cells completely lacking dCK expression were cultured in cytarabine-free medium for up to 8 weeks, did not show any indication of regained dCK protein expression or activity (data not shown). Although the selection pressure was merely sustained and not increased over the 21 days separating the Z138-CytES0 cells from Z138-CytR21 cells (Fig. 1d), the dCK expression was not completely lost until 21 days of exposure to 0.3 μM cytarabine. The expression of dCK was validated through TaqMan probe-based RT-PCR, where a significant down-regulation of dCK could be confirmed in both Z138-CytES and Z138-CytR21 compared to Z138-CytNS. No significant change in dCK mRNA levels was seen comparing cells grown with (CytR) or without cytarabine (CytES21) for 21 days beyond ES cells (Fig. 3). Based on this data, we draw the conclusion that the strongest association between cytarabine resistance and dCK is on protein level.

The results show that complete loss of dCK is crucial for cytarabine resistance and that cells adapt gradually, with initial increased viability and later restored/increased proliferation when exposed to a fixed concentration of cytarabine.

### Re-introduction of dCK in CytR cells restore sensitivity to high concentrations of cytarabine

To validate the direct effect of dCK on resistance, a transient re-introduction of dCK in resistant cells was performed. DCK cDNA (see Additional file 1) and a control vector containing GFP were introduced via nucleofection. Cell viability was 80–85% 24-48 h after nucleofection of both control and dCK plasmid (data not shown).

**Fig. 4:**
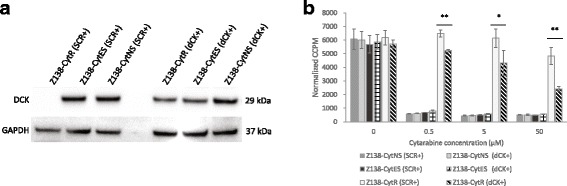
Assessment of the effects of re-introduction of dCK into resistant cells. **a** Western blot analysis of dCK proteins in Z138 sub-clones, 48 h after introduction of ORF-plasmid via nucleofection. GAPDH was used as loading control. **b** Normalized proliferation for Z138 cells grown in cytarabine containing medium, measured by incorporation of [methyl-14C]-thymidine 48 h after introduction of ORF-plasmid via nucleofection. Each data point represents a mean value of triplicates and error bars show SD. * = *p* < 0.05 and ** = *p* < 0.01, using students unpaired t-test

Protein expression was restored to levels correlating to Z138-CytNS cells (Fig. 4a). Z138-CytR cells where dCK expression had been transiently re-introduced (dCK+) were more sensitive to high concentrations of cytarabine, but the effect was less prominent for concentrations below 50 μM (Fig. 4b). This may be due to that the transfection efficiency measured after 24 h was around 65–80% (data not shown) and thus resistant clones, lacking dCK, remained.

### Gene expression analyses reveals major changes in transcription during adaptation, and the key protein SPIB related to development of resistance

**Fig. 5:**
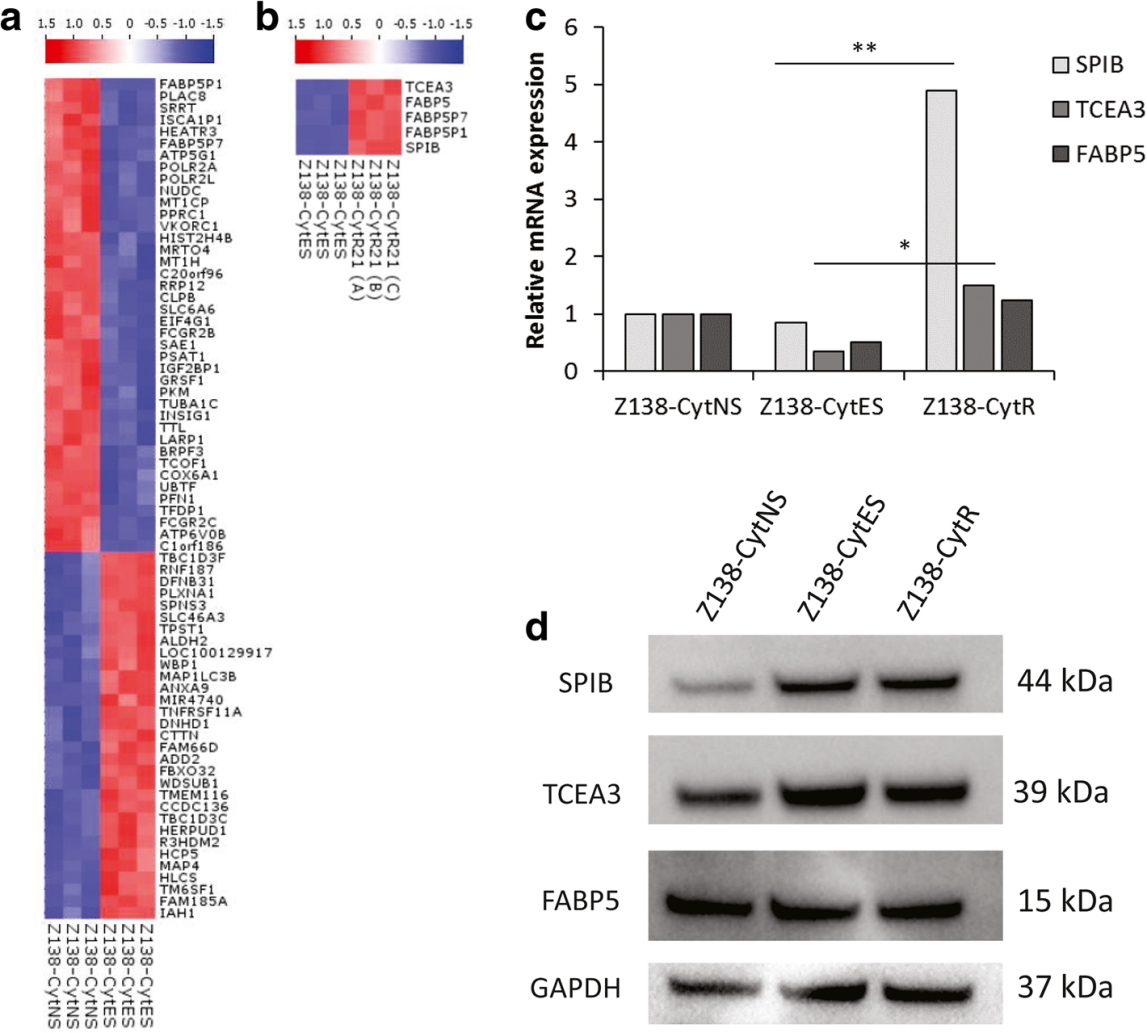
Heat maps of differentially expressed genes and assessment of their relative expression on qPCR and Western blot. Gene signatures associated to (**a**) adaptation (high viability), and (**b**) resistance (sustained proliferation) upon cytarabine exposure were identified by two-group comparison (q < 0.05) of Z138-CytNS vs. Z138-CytES, and Z138-CytES vs. Z138-CytR21, respectively. Red and blue colors indicate up- and down-regulated genes, respectively. **c** Assessment of relative SPIB, TCEA3 and FABP5 mRNA expression in Z138-CytNS, Z138-CytES and Z138-CyR cells. The relative mRNA expressions were assessed in the different Z138 subclones using TaqMan probe based RT-PCR and revealed up-regulation of all three genes for Z138-CytR cells compared to Z138-CytNS cells, although the difference was most prominent for SPIB. The data are normalized to the S18 reference gene, and the reference sample Z138-CytNS for each gene. * = *p* < 0.05 and ** = *p* < 0.01, with statistical significance determined using the Holm-Sidak method, with alpha = 0.05. Each row was analyzed individually, without assuming a consistent SD. **d** Representative (*n* = 3) western blot analysis of SPIB, TCEA3 and FABP5 proteins. GAPDH was used as loading control

To molecularly investigate resistance development, gene expression analysis was performed with resistant (Z138-CytR21) and control cells, from three different time-points (Z138-CytNS, Z138-CytES, Z138-CytES21 (CTR)). The microarray data was analysed in order to identify unique genes and/or gene signatures associated to (i) adaptation, or (ii) resistance. Upon two group comparisons of cytarabine naïve sensitive (Z138-CytNS) and cytarabine exposed sensitive (Z138-CytES) cells, 68 genes (Fig. 5a, Additional file 2: Table S3) were found as significantly (q < 0.05) deregulated and associated to the adaptation with high viability in presence of 0.2 μM cytarabine. Further analysis revealed that only minor transcriptional changes co-occur with the later phase of resistance development (Z138-CytES compared to Z138-CytR21). In total, 3 genes; FABP5, SPIB and TCEA3 (Fig. 5b, Additional file 2: Table S3) were found to be significantly (q < 0.05) deregulated. The upregulation of genes between Z138-CytES and Z138-CytR could be confirmed with TaqMan probe based RT-PCR (Fig. 5c), but both here and on protein level, SPIB showed the highest increase of expression in Z138-CytR compared to Z138-CytNS cells (Fig. 5d). In contrast to the mRNA, SPIB protein show increased level already in the Z138-CytES cells.

### Cytarabine resistant cells show increased levels of NF-κB and IκBα

**Fig. 6:**
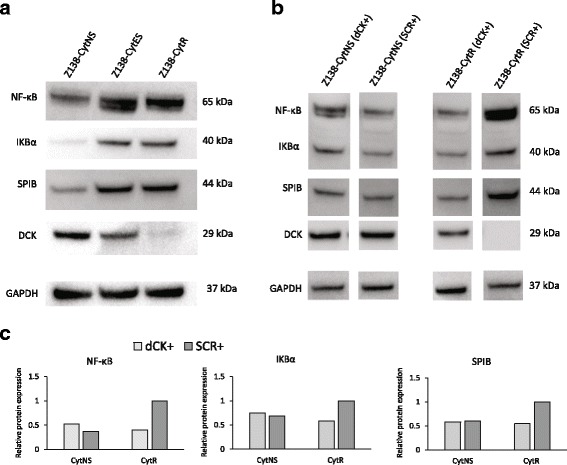
Assessment of NF-κB proteins in Z138-CytES and Z138-CytR cells compared to Z138-CytNS cells and with dCK re-expressed. **a** Due to the known connection between SPIB and the NF-κB pathway, protein expression of NF-κB and IKBα was assessed with western blot. GAPDH was used as loading control. **b** Western blot analysis of NF-κB, IKBα and SPIB protein levels in cells 48 h after transfection with ORF plasmid containing dCK. GAPDH was used as loading control. **c** Relative protein expression in the samples, normalized against GAPDH and with the Z138-CytR control sample set to 1 for each protein. Both western blot and calculated relative protein expression are representative figures of three biological replicates

MCL is known to be dependent on constitutive activation of NF-κB, and as SPIB has been reported to regulate this pathway in other lymphomas [22] we investigated the activity of the pathway in the different subclones. Increased activity in the resistant cells was confirmed through western blot which showed major increase in both total NF-κB and IκBα in Z138-CytR cells (Fig. 6a). Of note, re-introduction of dCK into resistant cells using transient overexpression, decreased the protein levels of SPIB, NF-κB and IKBα back to the original levels of untreated cells (Fig. 6b-c), indicating that dCK not only affect conversion of the pro-drug to active substance but also affect molecular pathways of importance for proliferation, differentiation and apoptosis.

### Knock-down of SPIB partly restores the sensitivity towards high doses of cytarabine in resistant cells

**Fig. 7:**
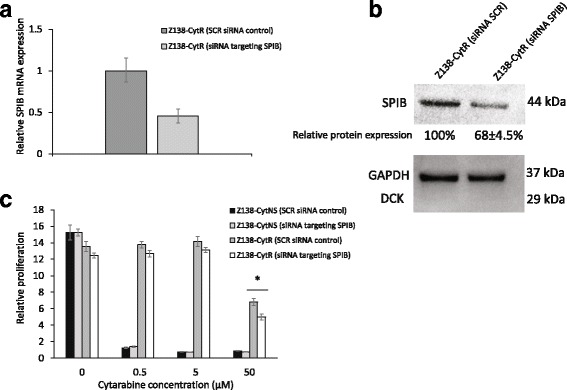
Effects of SPIB knock-down on resistant cells. **a** Conformation of partial silencing of SPIB on gene level. The relative mRNA expression of SPIB was assessed in the cells using TaqMan probe based RT-PCR and revealed a knock of gene expression with 50% compared to un-knocked control cells. The data are normalised to the S18 reference gene. **b** Protein levels of SPIB measured with western blot 48 h after transfection with siRNA. GAPDH was used as loading control. Representative figure of three biological replicates. **c** Normalised proliferation for Z138 cells grown in cytarabine containing medium, measured by incorporation of [methyl-14C]-thymidine 72 h after knock of SPIB using siRNA. Each data point represents a mean value of triplicates and error bars show SD. * = *p* < 0.05, using students unpaired t-test

To further understand the identified relation between SPIB upregulation and cytarabine resistance, SPIB was partly knocked-down using siRNA. SPIB mRNA decreased about 50% compared to the control 48 h after transfection (Fig. 7a). Western blot analysis revealed that protein levels were consistent with the decrease on mRNA level, with almost 40% less expression in knocked-down cells compared to control (Fig. 7b). This partial silencing decreased the proliferation of resistant cells with about 25% compared to the control cells in 50 μM cytarabine (Fig. 7c), but dCK protein levels were not restored. This indicates that SPIB does not directly regulate dCK.

### Z138-CytR cells are cross-resistant to other nucleoside analogues but sensitive to several clinically relevant drugs

**Fig. 8:**
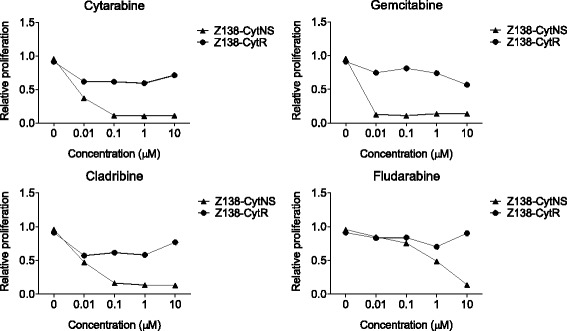
Evaluation of cross-resistance to common nucleoside analogues. Assessment of Z138-CytNS and Z138-CytR cell proliferation after treatment of cells for 48 h with different concentrations of four different nucleoside analogues, including cytarabine, gemcitabine, cladribine and fludarabine. The data are normalized to the 0-h time-point, and visualized as relative to the DMSO vehicle control. Each data point represents mean of three independent experiments

To investigate the potential cross-resistance of Z138-CytR cells to other cytostatic compounds, a library of 40 chemotherapeutic agents was used to assess proliferation upon exposure to the substances at four different concentrations. The relative sensitivity of cytarabine naïve sensitive (Z138-CytNS) and cytarabine resistant cells (Z138-CytR) was compared (Additio0nal file 1: Figure S3A-B). Results show that Z138-CytR, were cross-resistant to all nucleoside analogues evaluated (cladribine, fludarabine and gemcitabine), defined as < 50% reduction in proliferation, compared to the DMSO control, at any of the concentrations used (Additional file 2: Figure S3B, filled dark bars). The resistance to cladribine, fludarabine and gemcitabine was confirmed in separate experiments with a wider concentration range (Fig. 8). Wild type cells were sensitive to gemcitabine and cladribine, and thus cross-resistance is detected already at low concentrations (0.01 and 0.1 μM respectively) while reduction of proliferation after exposure to fludarabine was seen at or above 1 μM.

Interestingly, comparing the response of cytarabine naïve sensitive and cytarabine resistant cells to treatment with 1 and/or 10 μM chemotherapeutic library, eleven substances were identified to have an anti-proliferative effect (defined as > 50% reduction in proliferation compared to DMSO control) on both Z138-CytNS and Z138-CytR cells (Additional file 2: Figure S3, empty bars). These include topotecan HCl, teniposide, oxaliplatin, vincristine, paclitaxel, mitoxantrone hydrochloride, etoposide, docetaxel, cerubidine and doxorubicin and are thus of interest for treatment of patients who relapse on cytarabine-containing regiments.

### Histone deacetylase inhibitors have a stand-alone effect in cytarabine resistant cells and show a weak tendency to restore cytarabine sensitivity

To evaluate the stand-alone effect of various epigenetic drugs, a library of 43 epigenetic substances was used to assess sensitivity in cytarabine resistant cells. Eleven of the tested reagents from the epigenetic library had a stand-alone effect (defined as > 50% reduction in proliferation compared to DMSO control) on Z138-CytR cells at 10 μM (Additional file 2: Figure S4A right, filled dark bars). Of note, a majority of the effective compounds were histone deacetylase (HDAC) inhibitors.

For further validation, six compounds (apicidin, M-344, oxamflatin, scriptaid, trichostatin A and vorinostat) effective already at 1 μM (Additional file 2: Figure S4A left, filled dark bars) were selected (BML-281 and NSC-3852 were not commercially available in larger quantities). An additional HDAC inhibitor, not included in the library (belinostat) and a negative DMSO control were also included in the further evaluation. All substances, except for the negative control exhibited an inhibitory effect on Z138-CytR cell proliferation at concentrations above 1 μM (Additional file 2: Figure S4-B).

**Fig. 9:**
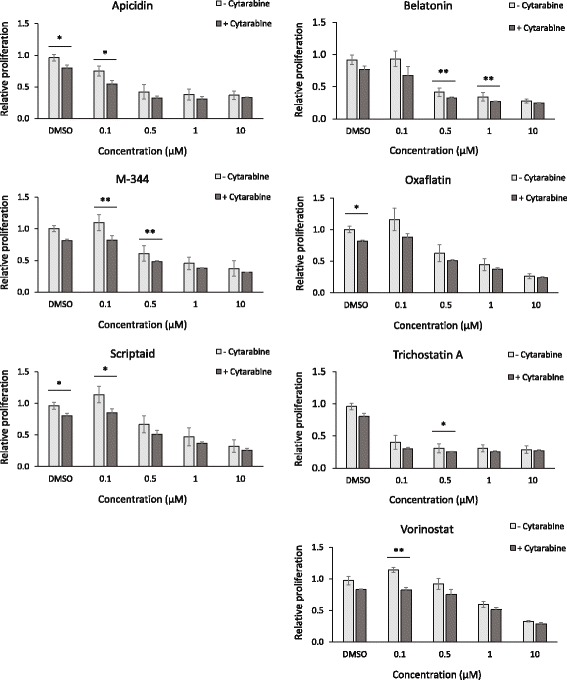
Assessment of epigenetic drug-mediated sensitization of cytarabine resistant cells. Proliferation of Z138-CytR cells exposed to different concentrations of six epigenetic drugs for 6 hours, prior to addition of 50 μM cytarabine. Several epigenetic drugs showed a tendency to re-sensitize Z138-CytR to cytarabine, at the lowest concentration (0.1 μM). Each data point represents mean relative proliferation ± SEM of three independent experiments. * = *p* < 0.05 and ** = *p* < 0.01, using students unpaired t-test. The data, collected 30 h after treatment, are normalized to the 0-h time-point and the R10 medium control (+/− cytarabine)

Similar effects were observed for the cytarabine naïve sensitive (Z138-CytNS) cells (Additional file 2: Figure S5). Based on these observations, additional experiments were performed in order to investigate the potential sensitizing effect on cytarabine resistant cells. Z138-CytR cells were exposed to a wide range of concentrations for 6 h prior to addition of cytarabine. A number of the HDAC inhibitors showed tendencies to induce cytarabine sensitivity in Z138-CytR cells at their lowest concentrations (0.1–0.5 μM), but for two of them, the difference between growth with or without cytarabine was significant also for the DMSO control, indicating that these results may be false positive (Fig. 9). Taken the effect of cytarabine alone into account, belatonin, M-344, trichostatin A and vorinostat stand out as possible sensitizers. A longer exposure time than 6 h was not technically feasible, which may have limited the impact of the epigenetic drugs.

Previous attempts to re-sensitize cells with resistance to nucleoside analogues include treatment with melatonin, an indolamine that overcome resistance to clofarabine by increasing expression of dCK [23]. However, in our system co-culture with melatonin or hydralazine could not re-sensitize cells to cytarabine after dCK down-regulation (data not shown).

### Resistant cells show slightly increased sensitivity towards lenalidomide and ibrutinib

In our search for drugs effective against cytarabine-resistant cells, both a targeted and library approach was used. It is well known that both ibrutinib and lenalidomide targets the NF-κB pathway, and thus the sensitivity towards these drugs was assessed. Z138-CytR and Z138-CytES cells showed significantly increased sensitivity towards 50 μM ibrutinib compared to Z138-NS, although at such high concentrations, proliferation is severely impaired for all subclones (Additional file 2: Figure S6A). The sensitivity towards lenalidomide was also significantly increased for both Z138-CytR and Z138-CytES cells compared to Z138-NS (Additional file 2: Figure S6B), although at a low magnitude.

### Bortezomib treatment prevents resistance development

**Fig. 10:**
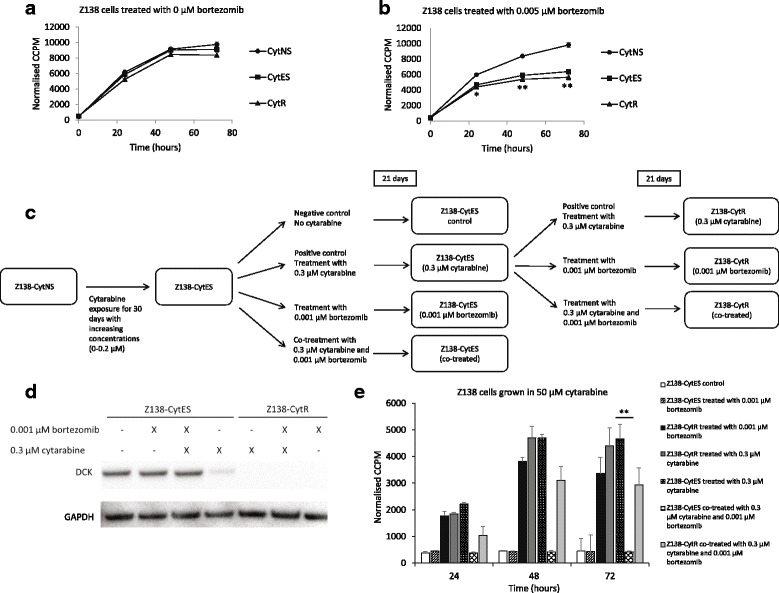
Protein expression of dCK and proliferation of Z138 sub-clones after co-treatment with bortezomib. Growth of cytarabine naive sensitive (Z138-CytNS) cells, cytarabine exposed sensitive (Z138-CytES) cells and cytarabine resistant (Z138-CytR) cells in presence of (**a**) no drug or (**b**) 0.005 μM bortezomib after 24–72 h, measured by incorporation of [methyl-14C]-thymidine. Each data point represents a mean value of triplicates ± SEM. * = *p* < 0.05 and ** = *p* < 0.01, using students unpaired t-test. According to the established model, Z138-CytES cells develop full resistance against cytarabine after 21 days of exposure to 0.3 μM cytarabine. **c** Recap of the model from Fig. 1, introducing co-treatment with 0.3 μM cytarabine and 0.001 μM bortezomib, both during resistance development and once resistance have been established. **d** Western blot analysis of dCK in Z138 sub-clones after 3 weeks of treatment with 0.3 μM cytarabine or co-treatment with 0.3 μM cytarabine and 0.001 μM bortezomib. GAPDH was used as loading control. Representative figure of three biological replicates. **e** Growth of cytarabine naive sensitive (Z138-CytNS) cells, cytarabine exposed sensitive (Z138-CytES) cells and cytarabine resistant (Z138-CytR) cells in presence of 50 μM cytarabine measured by incorporation of [methyl-14C]-thymidine. Each data point represents a mean value of triplicates ± SEM. * = *p* < 0.05 and ** = *p* < 0.01, using students unpaired t-test

Bortezomib acts on the NF-κB pathway by preventing the degradation of IκBα. We could confirm that Z138-CytR cells were more sensitive to bortezomib concentrations above 0.005 μM compared to Z138-NS (Fig. 10a-b) while lower concentrations did not affect proliferation (Additional file 2: Figure S1B). We hypothesized that continued exposure to the drug would alter the possibility of the cells to acquire resistance to cytarabine, either through the decreased degradation of IκBα or through changes in proteins regulating dCK on the protein level. To assess this, the cell model was used to co-treat cells with cytarabine and a low concentration of bortezomib during the 21 days expected for resistance to occur (Fig. 10c). The concentration was selected to avoid effect on proliferation. Co-treatment with 0.001 μM bortezomib and 0.3 μM cytarabine prevented the expected down-regulation of dCK (Fig. 10d) and as a result the Z138-CytES cells remained sensitive towards cytarabine (Fig. 10e). Z138-CytR cells remained dCK negative after long-term exposure (3 weeks) to bortezomib, and thus retained their resistance to cytarabine. To assess whether resistance was prevented or merely delayed by prolongated proliferation, relative cell number was monitored throughout the co-treatment study, and no difference in growth rate could be detected for Z138-CytR cells co-treated with bortezomib compared to those grown in cytarabine alone (Additional file 2: Figure S1B).

### Moderate to high expression of dCK is common in MCL diagnostic samples and corresponds well to initial successful response to cytarabine-containing treatment

**Fig. 11:**
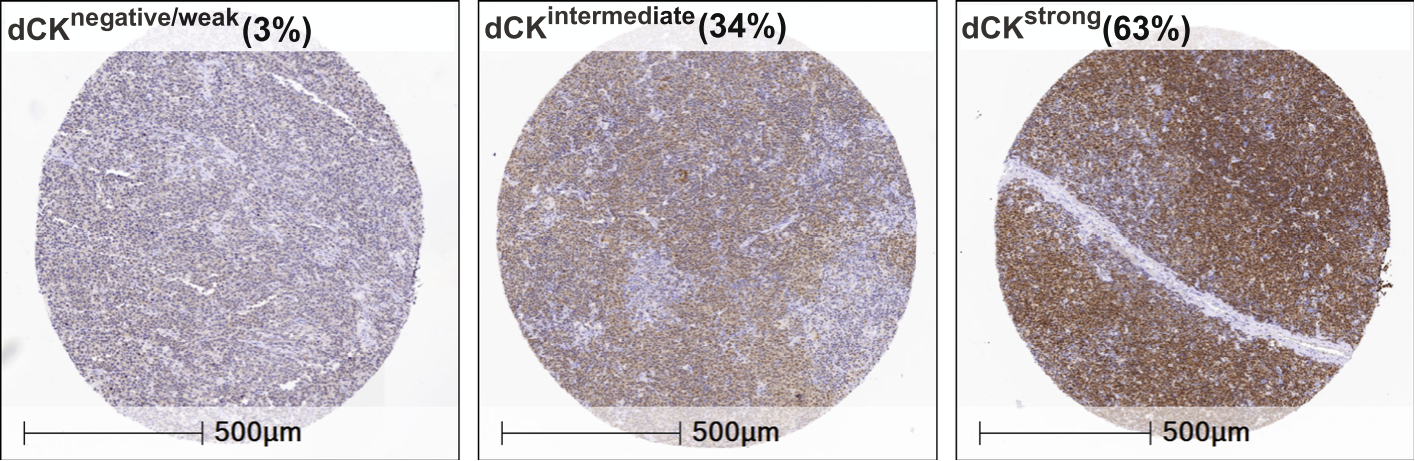
Representative immunostainings of dCK. The immunohistochemistry panel shows representative figures of negative/weak, intermediate, and strong dCK staining assessed by digital scoring using HALO™

Protein levels of dCK were stained for and automatically scored in 124 diagnostic samples from patients from the MCL2/3 cohort. Information on intensity and frequency of positive cells could be combined to define dCK strong (> 55% positive cells, *n* = 78), dCK intermediate (< 55% positive cells, *n* = 42) and dCK negative samples (> 90% negative cells, *n* = 4) (Fig. 11, and Additional file 2: Table S2). Thus, 120 of 124 patients (97%) had extensive dCK expression in the diagnostic sample, corresponding to the overall initial high response to treatment with cytarabine-containing regiments with 95% of the patients in the MCL2/3 cohort are progression free 6 months after inclusion to trial. As the number of dCK negative/weak patients where low, it was not statistical feasible to evaluate differences in survival.

## Discussion

Resistance to cytarabine is a significant clinical problem, as this agent is part of the backbone of treatment in a wide range of malignancies, including MCL. Thus, ways of predicting and possibly prevent cytarabine resistance are needed. The aim of the present study was to further explore the mechanisms of cytarabine resistance in MCL, to identify drugs suitable for treatment of patients with relapsed/recurrent disease and to identify preventive measures. To address these important clinical questions, we have established a unique MCL resistant model, based on the cell line Z138, in which cytarabine resistance repetitively in a timely and molecularly reproducibly manner can be induced. This cellular model allows for the first time resistance to be studied over time, and thus constitute an important research tool, despite the obvious drawback of being based on a single cell line It is known that dCK which is a key enzyme in converting cytarabine to active drug is dependent on functional p53 [24]. To ensure that the developed resistance was not caused by introduction of TP53 mutations due to cellular stress and selection pressure, the resistant cells were sequenced and found to contain only TP53 WT alleles.

Transportation and activation of cytarabine is mediated by several classes of transporters and activating enzymes, such as ENT [11] and dCK [12], as described in more detail elsewhere [25]. Using molecular profiling, we show that in our model, down-regulation of ENT1 is associated with adaptation to cytarabine exposure, but no further decrease is seen upon final development of resistance. Thus, in our model, ENT1 is not the limiting factor for resistance development, but rather a stand-by effect related to exposure to cytarabine. Also, previous studies using MCL cell lines show that there is a correlation between the expression of ENT1 and sensitivity to nucleoside analogues such as gemcitabine [26]. In childhood AML patients, sensitivity towards nucleoside analogues could be linked to ENT1 mRNA levels, with decreased levels of ENT1 in cytarabine resistant compared to sensitive patients [27]. However, it is not clear from these studies whether the down-regulation of ENT1 is a bystander effect, as our results indicate, or the limiting factor for drug sensitivity.

In the current study, we show that the final step of resistance development is associated with a complete down-regulation of the dCK protein. To investigate if the cellular levels of dCK protein is regulated at the transcriptional level, we performed gene expression analysis followed by RT-PCR validation. We show that dCK mRNA is down-regulated upon exposure and adaptation to cytarabine, but with minor additional down-regulation upon resistance development, indicating that post-translational modification may be involved in the final steps of resistance development. The relation between the dCK mRNA level and enzymatic activity has been explored previously. For example, using gemcitabine treatment in tumours of different origin such as pancreas and lung, it was shown that resistance only is predicted by dCK activity and protein level, and not by dCK mRNA level [28], in agreement with our data. DCK enzymatic activity is controlled by phosphorylation at Ser-74 [29], and dephosphorylation decreases enzyme activity [30]. A more active mutant of dCK, with a 10,000-fold increased sensitivity to nucleoside analogues has been created and could be of interest for suicide gene approaches [10, 31]. Of note, in our hands forced overexpression of dCK had a direct effect on sensitivity to cytarabine in our model, indicating that protein levels govern resistance but that post-translational modification adjust the level of resistance.

To further molecularly characterize the two distinct phases of (i) cytarabine adaptation and (ii) resistance development, global gene expression analysis was performed. Analysis reveals that major changes (68 genes) in gene expression could be associated with the initial adaptation to growth in cytarabine containing media. In contrast, only a few genes (FABP5P7, FABP5P1, FABP5, SPIB and TCEA3) showed expression changes upon final resistance development. Among those genes, SPIB showed major changes on the protein level. It is known from previous reports that SPIB is frequently overexpressed in diffuse large B cell lymphomas (DLBCL), and is a poor prognostic factor [22]. Of note, SPIB and IRF4 have been shown to amplify NF-ĸB signalling by transactivating CARD11 [32]. SPIB has not previously been described in relation to MCL and the connection to increased NF-ĸB activity in our model is of major interest. NF-ĸB is highly active in MCL and is considered to be a key feature for the aggressiveness of MCL [33]. Constitutively active NF-κB in MCL may be caused by different factors such as, chronic activation of the B cell receptor (BCR) or mutations in the Toll like receptor (TLR) signalling pathway. When the activation is a result of somatic mutations on inhibitors of the alternative pathway, the activation is unaffected by BCR inhibitors [4]. To pursue if the increased aggressiveness of the resistant cells could be related to enhanced NF-ĸB activity, related proteins were investigated. Analysis showed that the resistant cells have elevated levels of both NF-ĸB total protein as well as IκBα, indicating increased engagement of the pathway in dCK^negative^/SPIB^high^ cells exhibiting resistance to cytarabine. Of major interest, forced overexpression of dCK led to decreased SPIB and NF-ĸB levels showing for the first time that dCK not only have an impact on the conversion of pro-drugs into active drugs, but also contribute to resistance through direct transcriptional control of pathways involved in proliferation and apoptosis. DCK as a key-driver was further pin-pointed by the fact that knock-down of SPIB did not affect dCK or NF-ĸB levels, although cytarabine resistance was slightly affected. Thus, SPIB does not regulate dCK levels but is likely associated with increased resistance through other means.

As NF-ĸB seem to play a major role in resistance we assessed if drugs known to be able to affect NF-ĸB activity, such as the immune-modulating drug lenalidomide [34] and the Bruton tyrosine kinase (BTK) inhibitor ibrutinib [35], had increased activity in the resistant cells. However, it was only at high concentrations of lenalidomide and Ibrutinib that the resistant cells had a significant increased sensitivity to cytarabine treatment.

In order to identify clinically relevant drugs effective in cytarabine resistant cells, a library of chemotherapeutic compounds was used. Eleven drugs, some predominantly used in treatment of solid cancers, and to less extent in treatment of leukemias and lymphomas, showed strong anti-proliferative effect on cytarabine resistant cells. Interestingly, four of the eleven drugs have already been considered in treatment of MCL, either as part of standard therapy (doxorubicin and vincristine) or as part of clinical trials including refractory MCL (mitoxantrone [36] and etoposide [37]). Also previous studies on cytarabine resistant leukaemia cells show that such cells are sensitive to vincristine and mitoxantrone [38], supporting the clinical usefulness of these drugs for treatment of cytarabine-resistant disease. Novel drugs that may have a clinical role for treatment of cytarabine-resistant disease included topotecan HCl, teniposide, oxaliplatin, paclitaxel, docetaxel and cerubidine.

As expected, cytarabine resistant cells showed cross-resistance to other nucleoside analogues, including gemcitabine, cladribine and fludarabine, emphasizing the specificity of our model, development of nucleoside analogue-resistant cells and not a general increased drug resistance [12, 14].

It has been proposed that the down-regulation of dCK is mediated by epigenetic mechanisms [39, 40] and in order to evaluate if epigenetic drugs have a potent (i) stand-alone effect or potentially, (ii) a sensitizing effect, a library with epigenetic compounds was evaluated. Of interest, most of the compounds that showed inhibitory effect on cytarabine resistant cells were HDAC inhibitors. The potential of these compounds to re-sensitize resistant cells to cytarabine was assessed through pre-incubation prior to cytarabine exposure. Some of the compounds showed a minor tendency to increase cytarabine sensitivity after pre-treatment with low concentrations of HDAC inhibitors. Potentially, a longer pre-treatment would have generated a larger impact on cytarabine resistance. HDAC inhibitors generally have low toxicity and although further pre-clinical studies are needed, inclusion of such inhibitors in combination with cytarabine should be considered.

Our present data pinpoints the importance of dCK for resistance development, and thus ways of preventing and restoring dCK activity would have a major clinical impact for treatment of MCL patients. Several previous studies have used different approaches to increase dCK activity and thus revert cytarabine resistance. Among others, it has been shown that dCK was silenced through promoter DNA methylation, and that demethylation can restore dCK levels [39]. However, in our hands neither decitabine nor hydralazine resulted in restored sensitivity.

From our data, it is clear that dCK activity is governed by mechanisms at the protein level and we hypothesized that co-treatment with bortezomib, a proteasome inhibitor, could prevent down-regulation of dCK and development of resistance. Bortezomib, is a reversible proteasome inhibitor, primarily used in MCL as part of combinatorial therapy and approved for treatment of relapsed MCL [41]. Inhibition of the NF-κB pathway, leading to apoptosis, has been reported as a major mechanism of action for bortezomib [42] and that correlates well with our model, showing increased sensitivity to bortezomib (at concentrations above 0.005 μM) in resistant cells compared to sensitive. Interestingly, the effects of bortezomib were much more prominent than for both lenalidomide and ibrutinib. The difference may partly be explained by the fact that the targets of bortezomib affects proteins more directly involved in NF-κB activation and regulation. However, being a proteasome inhibitor the action of bortezomib is not specific for the NF-κB pathway and several other cellular targets and pathways may be affected. Co-treatment with bortezomib prevented the development of cytarabine resistance and constitute a very attractive complement to cytarabine treatment in clinical protocols. Proliferation was assessed and no difference in growth rate was observed between cells co-cultured with both bortezomib (at 0.001 μM) and cytarabine compared to cells treated with cytarabine alone, ruling out the possibility that the resistance was prevented due to decreased proliferation. To our knowledge, this is the first time that it has been shown that cytarabine resistance can be prevented, which is of major clinical importance. As bortezomib is an already approved drug, implementation into the clinic should be rapid. It should be noted however, that the concentrations used in our study is in the low-end of the bortezomib serum levels measured in patients treated within current dosing regiments [43]. Previous studies have demonstrated a synergy effect of bortezomib and cytarabine both in vitro and in individual case reports [44] and also as part of a small combinatorial therapy trial [45]. A clinical trial is currently ongoing, where bortezomib was combined with cytarabine for treatment of relapse patients, and results may shed light on the efficacy of this co-treatment [46]. Diagnostic/pre-treatment levels of dCK have in a previous study been shown to predict in vivo gemcitabine sensitivity in human tumour xenografts (pancreas, colon, ovarian cancer) [28]. Thus, using a cohort of patients treated with the combinatorial Nordic MCL2/3 protocol, where 95% of the patients are progression free ≥6 months after therapy including high-dose cytarabine, we investigated the diagnostic levels of dCK. The dCK levels were automatically scored to identify both frequency of positive tumour cells, but also intensity of nuclear dCK levels. Ninety seven percent of the patients showed high or intermediate expression of dCK, corresponding to the high clinical response to the cytarabine-containing combinatorial protocol. Thus, the number of dCK negative/weak patients were too few to evaluate impact on survival. Today, material from relapsed MCLs is not available but constitute an important work material to study to validate our finding of down-regulation of dCK and development of cytarabine resistance.

## Conclusions

With the aim to understand the molecular mechanisms of cytarabine resistance in MCL and identify substances that can be used to treat refractory and relapsed disease, we have established a cytarabine resistant in vitro model. This model has been used to show that cytarabine resistance occurs as a result of complete dCK down-regulation and that resistant cells show increased SPIB and NF-κB. We show for the first time that dCK not only is associated with phosphorylation of pro-drugs, but also directly affect SPIB and NF-κB levels in resistant cells, and thus have impact on pathways crucial for proliferation and resistance to apoptosis. The use of chemotherapeutic and epigenetic compound libraries has pin-pointed clinically relevant drugs that can be used to treat resistant disease. Of note, co-treatment with bortezomib prevented down-regulation of dCK and development of resistance and may have an immediate clinical impact on the treatment of MCL patients. The results that were based on a single cell line have generated hypothesis regarding how cytarabine-resistance may be avoided and targeted in the clinic, and should be further validation in clinical material such as collection of relapsed samples and samples from clinical studies with combined use of bortezomib and cytarabine.

## Additional files

Additional file 1: Supplementary material and methods. (PDF 198 kb)

Additional file 2: Supplementary data. (DOCX 1430 kb)

## Abbreviations

CNT: Concentrative nucleoside transporter
dCK: Deoxycytidine kinase
DLBCL: Diffuse large B cell lymphoma
ENT: Equilibrative nucleoside transporter
HDAC: Histone deacetylase
IκBα: Inhibitor of nuclear factor kappa B alpha
MCL: Mantle cell lymphoma
NF-κB: Nuclear factor kappa B
Z138-CytES: Z138-Cytarbine Exposed Sensitive
Z138-CytNS: Z138-Cytarbine Naïve Sensitive
Z138-CytR: Z138-Cytarbine Resistant
Z138-CytR21: Z138-Cytarbine Resistant 21 days
