# Supplementary material for: Bortezomib prevents cytarabine resistance in MCL, which is characterized by down-regulation of dCK and up-regulation of SPIB resulting in high NF-κB activity
Source: BMC Cancer. 2018 Apr 25;18:466. doi: 10.1186/s12885-018-4346-1 (PMC5918903; doi:10.1186/s12885-018-4346-1)

Supplementary Material and Methods

Combinatory treatment with lenalidomide and ibrutinib

Cells were seeded and treated with 0.3 µM cytarabine, 0.01 µM bortezomib or 0.1 µM lenalidomide, alone or in combination with each other. Duplicates from each concentration were counted in an automatic cell counter (CountessTM, Invitrogen) at each time point, and trypan blue exclusion method was used to monitor viability. Lysates for RT-qPCR and immunoblotting was sampled after 24 and 48 hours of growth. No significant differences could be observed in any of the sub clones and no synergy effects could be observed when combining lenalidomide and bortezomib (data not shown).

Vector Information DCK OmicsLink^{TM} Expression Clone (EX-C0081-M46)

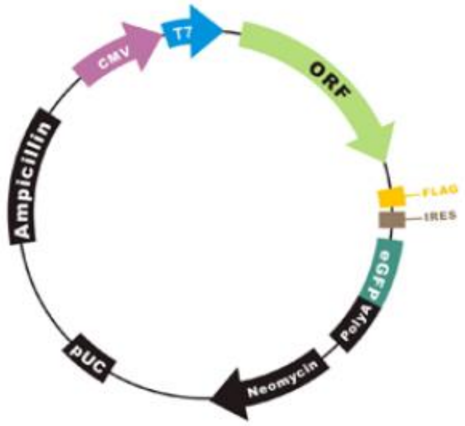

Supplement: Supplementary file 1 — Supplementary material and methods. (PDF 198 kb) [file 12885_2018_4346_MOESM1_ESM.pdf]
