# Supplementary material for: Bortezomib prevents cytarabine resistance in MCL, which is characterized by down-regulation of dCK and up-regulation of SPIB resulting in high NF-κB activity
Source: BMC Cancer. 2018 Apr 25;18:466. doi: 10.1186/s12885-018-4346-1 (PMC5918903; doi:10.1186/s12885-018-4346-1)
Supplement: Supplementary file 2 — Supplementary data. (DOCX 1430 kb) [file 12885_2018_4346_MOESM2_ESM.docx]

**Supplementary data**





**Figure S1. Proliferation and cell growth in untreated and treated cells**  A) Different subclones grown in cytarabine free medium. Each data point represents mean relative proliferation ± SD of nine independent experiments. The data, collected 24 and 48 hours after treatment, are normalized to the 0 hour time-point for each experiment and cell line. B) Relative cell count of the different sub clones during co-treatment experiment with bortezomib and cytarabine. Cells are grown in 0.001 µM bortezomib, with or without addition of 0.3 µM cytarabine. Each data point represents mean relative cell count ± SD of three independent experiments. The data, collected after 3 weeks of treatment, is normalized to the seeding density 48 hours earlier.

***
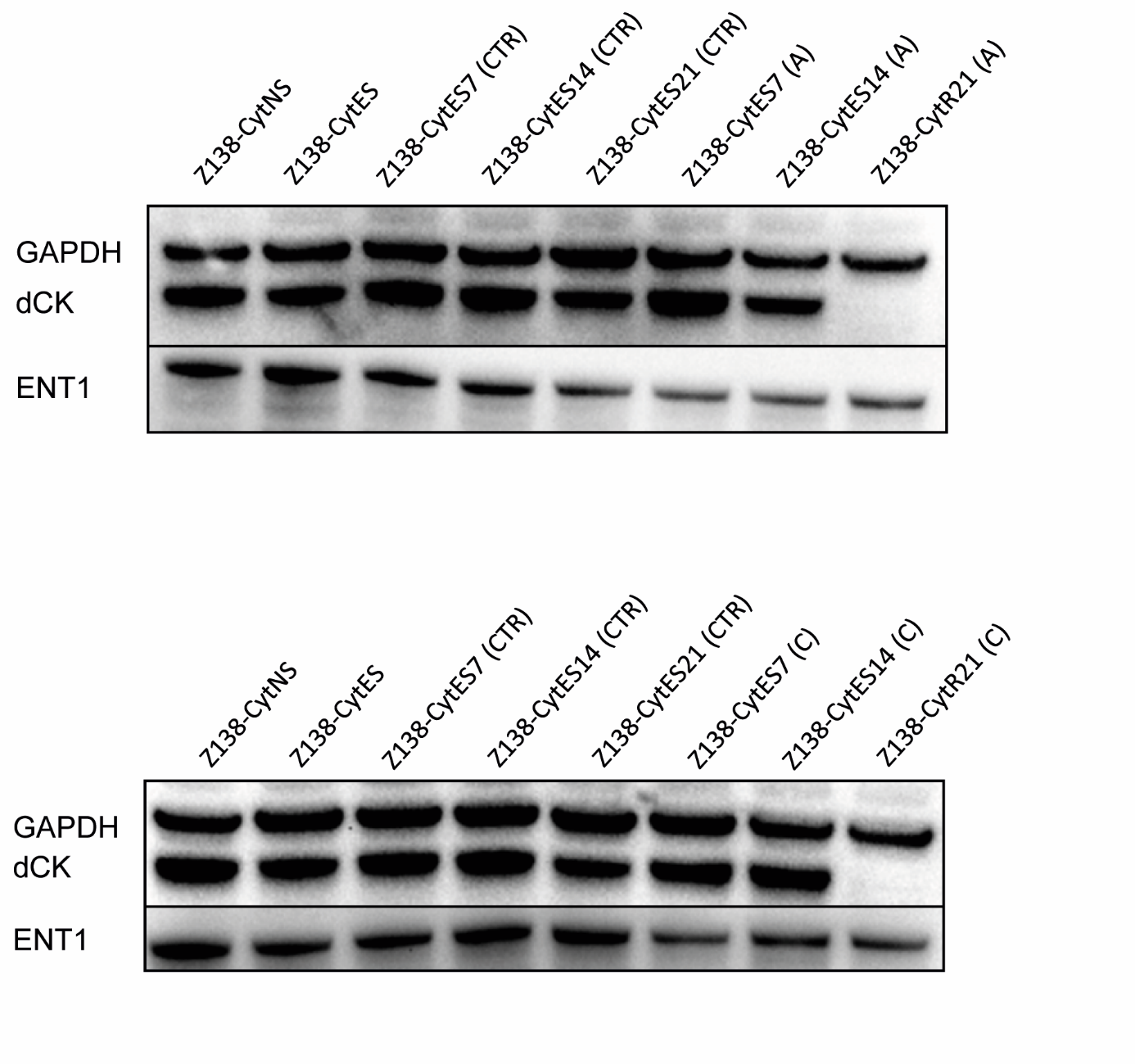
***

**Figure S2. Western blot analysis of dCK and ENT1.** Western blot analysis of indicated proteins in Z138 sub-clones. CytNS: cytarabine naive sensitive, CytES: cytarabine exposed sensitive, CytR21: cytarabine resistant, CTR: control, B: replicate B. GAPDH was used as loading control. These results are consistent with the third biological replicate presented in Fig 2.


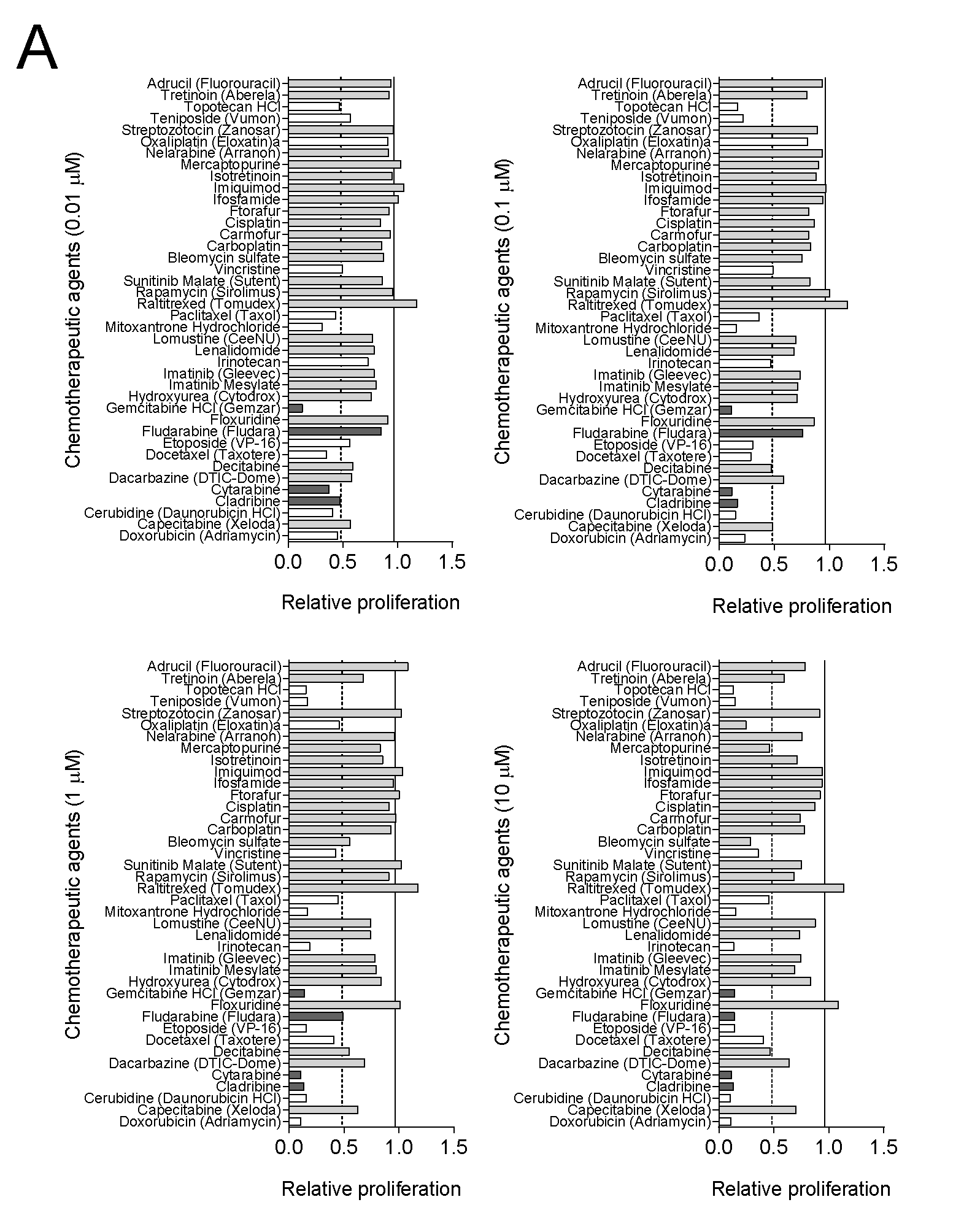


**
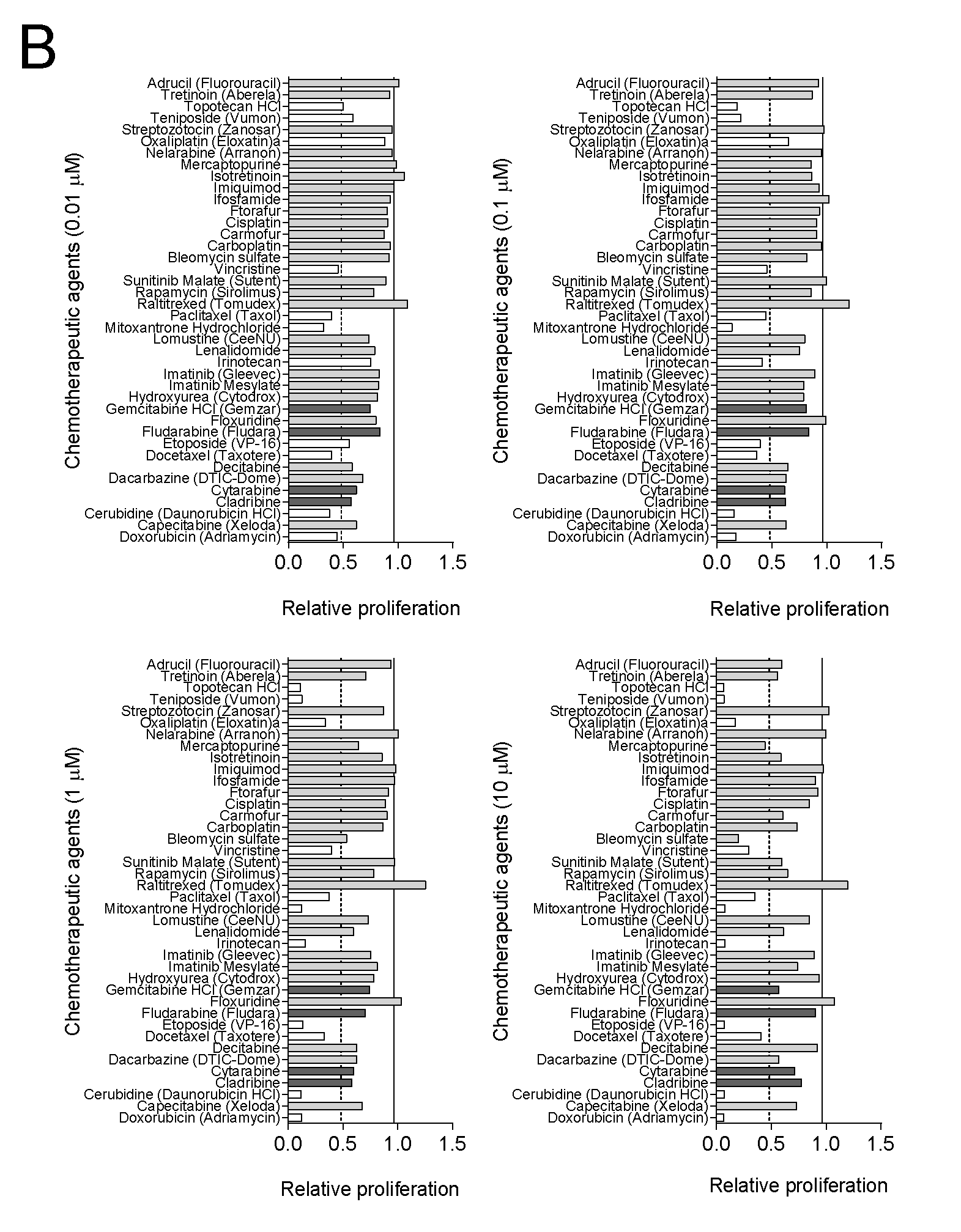
**

**Figure S3. Assessment of sensitivity to different chemotherapeutic drugs.** The relative proliferation of A) wild type Z138 cytarabine naïve sensitive (Z138-CytNS) and B) cytarabine resistant (Z138-CytR) cells was assessed after 48 hours of treatment with a chemotherapeutic library, at four different concentrations. Following cell proliferation, eleven drugs (empty bars) show at 1 and 10 µM a stand-alone anti-proliferative effect (defined as >50% reduction in proliferation compared to DMSO control) on both Z138-CytNS and Z138-CytR. Comparing Z138-CytNS and Z138-CytR, three substances (filled dark bars) show cross-resistance to cytarabine. The data are normalized to the 0-hour time-point, and visualized as relative to the DMSO vehicle control (full line). Dashed line: 0.5 × DMSO.


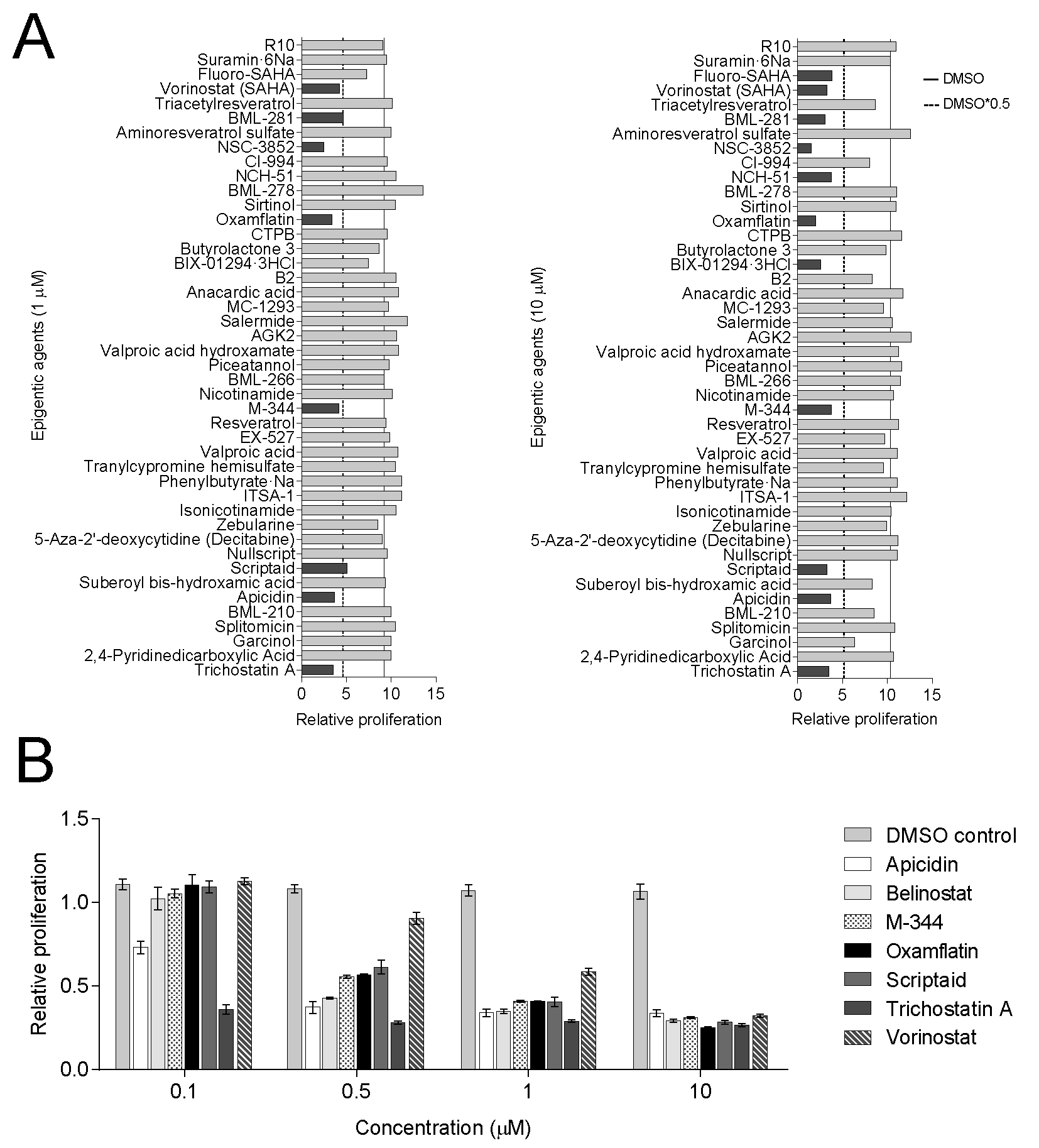


**Figure S4. Evaluation of Z138-CytR treatment response to epigenetic drugs**. A) Z138-CytR cells were treated for 24 hours with a library of epigenetic drugs (n=43). Following cell proliferation assessment, eleven drugs (filled dark bars) were showing, for at least one concentration, a stand-alone effect (≥ 50% reduction in proliferation compared to DMSO control) on Z138-CytR. The data is normalized to the 0-hour time-point. Full line represents the DMSO vehicle control, and dashed line half of the value of the DMSO control. B) Six of the eleven epigenetic drugs showing a stand-alone effect were available in larger quantities, and were further evaluated in Z138-CytR cells, including one additional drug (belinostat). Each data point represents mean relative proliferation ± SEM of three independent experiments. The data, collected 30 hours after treatment, are normalized to the 0-hour time-point and the DMSO vehicle control.


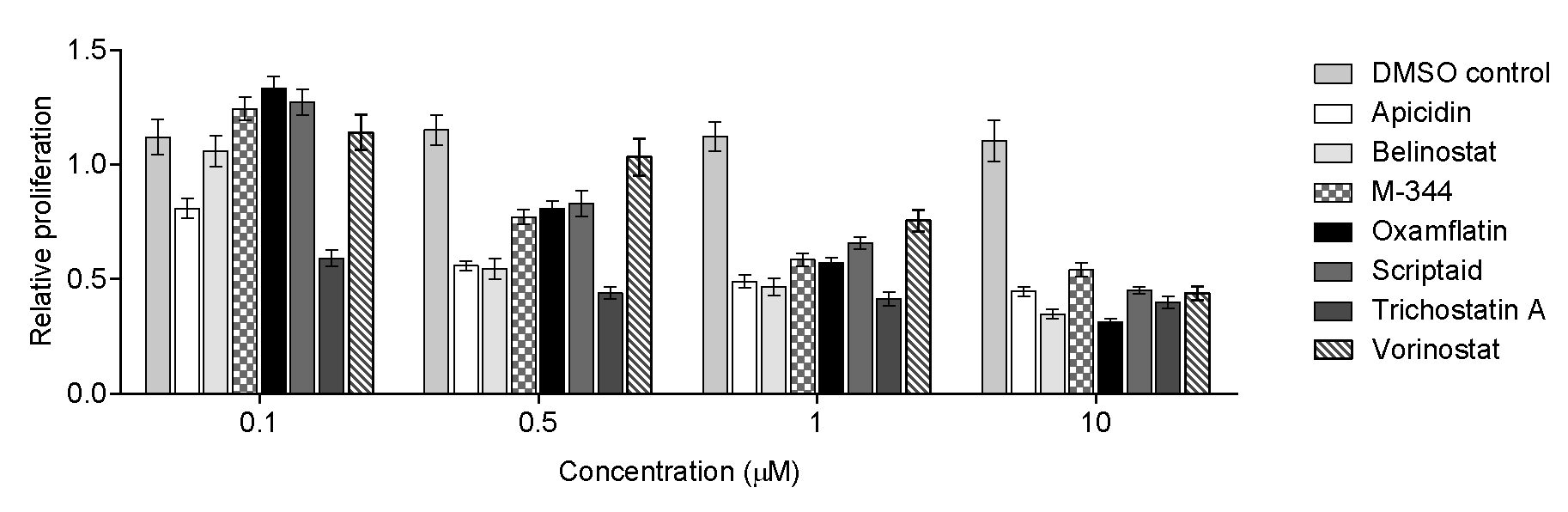


**Figure S5. Evaluation of Z138-CytNS treatment response to epigenetic drugs**. 7 epigenetic drugs that have shown a stand-alone effect in Z138-CytR cells were also tested on Z138-CytNS cells. Each data point represents mean relative proliferation ± SEM of three independent experiments. The data, collected 30 hours after treatment, are normalized to the 0-hour time-point and the DMSO vehicle control.


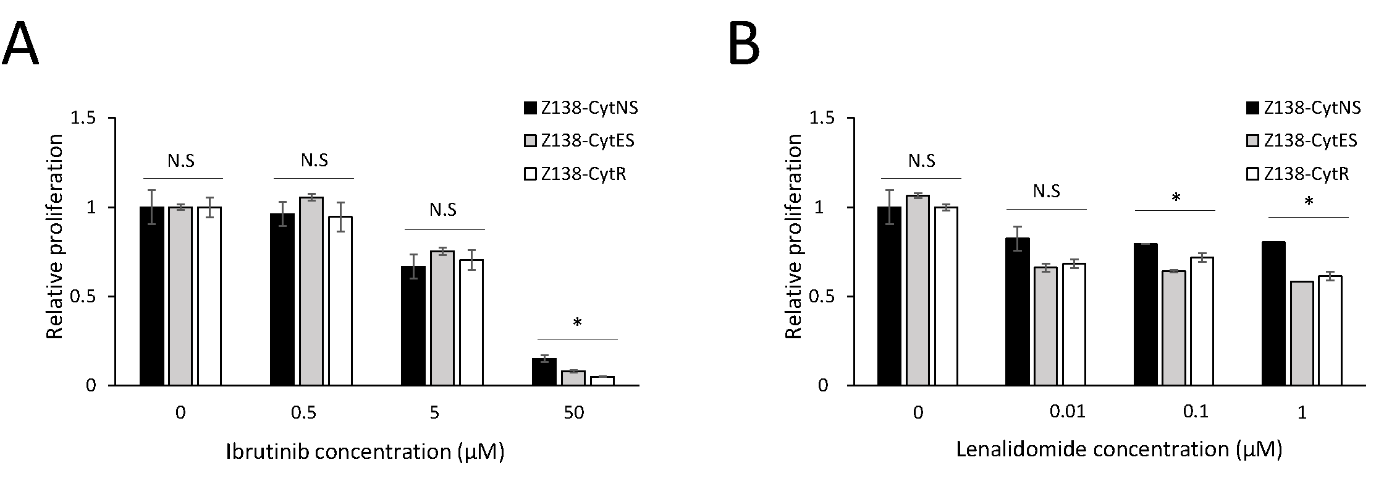


**Figure S6**. **Proliferation of Z138 sub-clones in ibrutinib or lenalidomide-containing medium**. Growth of cytarabine naive sensitive (Z138-CytNS) cells, cytarabine exposed sensitive (Z138-CytES) cells and cytarabine resistant (Z138-CytR) cells in presence of A) 0-50 µM of ibrutinib or B) 0-1 µM lenalidomide 48 hours after seeding, measured by incorporation of [methyl-14C]-thymidine. Each data point represents a mean value of triplicates and error bars show SEM. *= p ≤ 0.05, using students unpaired t-test. All significance is by comparison to Z138-CytNS cells.

**Table S1. Analysis settings in HALO^TM^ maintained throughout the dCK analyses.**

| Classifier | classifier_1704 |
| --- | --- |
| Class List | Tumor |
| Image Zoom | 1 |
| Nuclear Stain | 0.154,0.174,0.117 |
| Positive Stain 1 | 0.222,0.365, 0.447 |
| Positive Stain 2 | 0.0,0.0,0.0 |
| Stain 1 Localization | Nucleus |
| Stain 2 Localization | Cytoplasm |
| Minimum Tissue OD | 0.028 |
| Tissue Edge Thickness | 5.6 |
| Nuclear Contrast Threshold | 0.503 |
| Minimum Nuclear OD | 0.007 |
| Nuclear Size (Minimum, Maximum) | 3.275, 207.965 |
| Minimum Nuclear Roundness | 0 |
| Nuclear Segmentation Aggressiveness | 0.923 |
| Fill Nuclear Holes | False |
| Maximum Cytoplasm Radius | 5 |
| Stain 1 Min OD (Weak, Moderate, Strong) | 0.062,0.159,0.264 |
| Stain 2 Min OD (Weak, Moderate, Strong) | 0.112,0.287,0.547 |
| Stain 1 Mask | not set |
| Stain 2 Mask | not set |
| Output Image | Stain 1 Markup |
| Verbose Output | CSV |

**Table S2. dCK mean levels when divided into three groups with mean low, intermediate and high expression**

|  | Frequence positive cells (±SD) |
| --- | --- |
| dCK negative | 5.5±2.5 |
| dCK intermediate | 34.2±13.1 |
| dCK high | 88.6±8.1 |

**Table S3. Identification of gene signatures associated to initial adaption or final resistance to cytarabine exposure by two-group comparison (q<0.05) of Z138-CytNS versus Z138-CytES, and Z138-CytES versus Z138-CytR, respectively.**

| **Initial adaption (high viability)** | | | |
| --- | --- | --- | --- |
| **Gene Accession** | **Gene Symbol** | **Gene Description** | **Fold change** |
| ENST00000458175 | FABP5P7 | fatty acid binding protein 5 pseudogene 7 | -3,26 |
| NM_001130715 | PLAC8 | placenta-specific 8 | -2,98 |
| ENST00000420287 | FABP5P1 | fatty acid binding protein 5 pseudogene 1 | -2,92 |
| NM_201563 | FCGR2C | Fc fragment of IgG, low affinity IIc, receptor for (CD32) (gene/pseudogene) | -2,69 |
| NM_001007544 | C1orf186 | chromosome 1 open reading frame 186 | -2,67 |
| NM_001002273 | FCGR2B | Fc fragment of IgG, low affinity IIb, receptor (CD32) | -2,45 |
| NM_000937 | POLR2A | polymerase (RNA) II (DNA directed) polypeptide A, 220kDa | -2,15 |
| NM_001034077 | HIST2H4B | histone cluster 2, H4b | -1,91 |
| NM_015062 | PPRC1 | peroxisome proliferator-activated receptor gamma, coactivator-related 1 | -1,91 |
| NM_001002027 | ATP5G1 | ATP synthase, H+ transporting, mitochondrial Fo complex, subunit C1 (subunit 9) | -1,71 |
| NM_030813 | CLPB | ClpB caseinolytic peptidase B homolog (E. coli) | -1,70 |
| ENST00000379816 | MT1CP | metallothionein 1C, pseudogene | -1,69 |
| NM_004373 | COX6A1 | cytochrome c oxidase subunit VIa polypeptide 1 | -1,69 |
| NM_001206796 | PKM | pyruvate kinase, muscle | -1,68 |
| NM_058179 | PSAT1 | phosphoserine aminotransferase 1 | -1,67 |
| NM_001134367 | SLC6A6 | solute carrier family 6 (neurotransmitter transporter, taurine), member 6 | -1,59 |
| NM_001145713 | SAE1 | SUMO1 activating enzyme subunit 1 | -1,58 |
| NM_007111 | TFDP1 | transcription factor Dp-1 | -1,57 |
| NM_001098477 | GRSF1 | G-rich RNA sequence binding factor 1 | -1,57 |
| NM_021128 | POLR2L | polymerase (RNA) II (DNA directed) polypeptide L, 7.6kDa | -1,56 |
| NM_005022 | PFN1 | profilin 1 | -1,54 |
| ENST00000542811 | ISCA1P1 | iron-sulfur cluster assembly 1 homolog (S. cerevisiae) pseudogene 1 | -1,52 |
| NM_182922 | HEATR3 | HEAT repeat containing 3 | -1,51 |
| NM_016183 | MRTO4 | mRNA turnover 4 homolog (S. cerevisiae) | -1,48 |
| NM_001160423 | IGF2BP1 | insulin-like growth factor 2 mRNA binding protein 1 | -1,47 |
| NM_001039457 | ATP6V0B | ATPase, H+ transporting, lysosomal 21kDa, V0 subunit b | -1,45 |
| NM_001145114 | RRP12 | ribosomal RNA processing 12 homolog (S. cerevisiae) | -1,43 |
| NM_005542 | INSIG1 | insulin induced gene 1 | -1,41 |
| NM_080571 | C20orf96 | chromosome 20 open reading frame 96 | -1,40 |
| NM_001194946 | EIF4G1 | eukaryotic translation initiation factor 4 gamma, 1 | -1,40 |
| NM_001128852 | SRRT | serrate RNA effector molecule homolog (Arabidopsis) | -1,39 |
| NM_024006 | VKORC1 | vitamin K epoxide reductase complex, subunit 1 | -1,39 |
| NM_006600 | NUDC | nuclear distribution C homolog (A. nidulans) | -1,38 |
| NM_000356 | TCOF1 | Treacher Collins-Franceschetti syndrome 1 | -1,37 |
| NM_032704 | TUBA1C | tubulin, alpha 1c | -1,34 |
| NM_015315 | LARP1 | La ribonucleoprotein domain family, member 1 | -1,32 |
| NM_005951 | MT1H | metallothionein 1H | -1,31 |
| NM_153712 | TTL | tubulin tyrosine ligase | -1,27 |
| NR_045058 | UBTF | upstream binding transcription factor, RNA polymerase I | -1,27 |
| NM_015695 | BRPF3 | bromodomain and PHD finger containing, 3 | -1,17 |
| NM_001201372 | CCDC136 | coiled-coil domain containing 136 | 1,14 |
| NM_144666 | DNHD1 | dynein heavy chain domain 1 | 1,16 |
| NR_027425 | FAM66D | family with sequence similarity 66, member D | 1,17 |
| NR_039894 | MIR4740 | microRNA 4740 | 1,20 |
| NM_001039613 | IAH1 | isoamyl acetate-hydrolyzing esterase 1 homolog (S. cerevisiae) | 1,21 |
| NM_001135919 | SLC46A3 | solute carrier family 46, member 3 | 1,25 |
| NM_001128212 | WDSUB1 | WD repeat, sterile alpha motif and U-box domain containing 1 | 1,28 |
| NM_001193453 | TMEM116 | transmembrane protein 116 | 1,32 |
| NM_001184740 | CTTN | cortactin | 1,32 |
| NM_012477 | WBP1 | WW domain binding protein 1 | 1,33 |
| NM_003596 | TPST1 | tyrosylprotein sulfotransferase 1 | 1,33 |
| NR_036511 | LOC100129917 | uncharacterized LOC100129917 | 1,34 |
| NM_001185054 | ADD2 | adducin 2 (beta) | 1,37 |
| NM_000690 | ALDH2 | aldehyde dehydrogenase 2 family (mitochondrial) | 1,37 |
| NM_001145268 | FAM185A | family with sequence similarity 185, member A | 1,38 |
| NM_032258 | TBC1D3F | TBC1 domain family, member 3F | 1,40 |
| NM_001242463 | FBXO32 | F-box protein 32 | 1,43 |
| NM_001134364 | MAP4 | microtubule-associated protein 4 | 1,52 |
| NM_001001418 | TBC1D3C | TBC1 domain family, member 3C | 1,55 |
| NM_014925 | R3HDM2 | R3H domain containing 2 | 1,57 |
| NM_022818 | MAP1LC3B | microtubule-associated protein 1 light chain 3 beta | 1,61 |
| NM_001010990 | HERPUD1 | homocysteine-inducible, endoplasmic reticulum stress-inducible, ubiquitin-like domain member 1 | 1,74 |
| NM_000411 | HLCS | holocarboxylase synthetase (biotin-(proprionyl-CoA-carboxylase (ATP-hydrolysing)) ligase) | 1,75 |
| NM_182538 | SPNS3 | spinster homolog 3 (Drosophila) | 1,85 |
| NM_001144903 | TM6SF1 | transmembrane 6 superfamily member 1 | 1,89 |
| NM_003839 | TNFRSF11A | tumor necrosis factor receptor superfamily, member 11a, NFĸB activator | 2,05 |
| ENST00000393409 | PLXNA1 | plexin A1 | 2,07 |
| NM_015404 | DFNB31 | deafness, autosomal recessive 31 | 2,10 |
| NR_040662 | HCP5 | HLA complex P5 (non-protein coding) | 2,13 |
| NM_001010858 | RNF187 | ring finger protein 187 | 2,22 |
| NM_003568 | ANXA9 | annexin A9 | 2,42 |
| **Final resistance (sustained proliferation)** | | | |
| **Gene Accession** | **Gene Symbol** | **Gene Description** | **Fold change** |
| ENST00000458175 | FABP5P7 | fatty acid binding protein 5 pseudogene 7 | 3,43 |
| ENST00000420287 | FABP5P1 | fatty acid binding protein 5 pseudogene 1 | 3,12 |
| NM_001444 | FABP5 | fatty acid binding protein 5 (psoriasis-associated) | 1,84 |
| NM_001243998 | SPIB | Spi-B transcription factor (Spi-1/PU.1 related) | 1,69 |
| NM_003196 | TCEA3 | transcription elongation factor A (SII), 3 | 1,25 |
